# Supplementary material for: State-aware detection of sensory stimuli in the cortex of the awake mouse
Source: PLoS Comput Biol. 2019 May 31;15(5):e1006716. doi: 10.1371/journal.pcbi.1006716 (PMC6561583; doi:10.1371/journal.pcbi.1006716)
Supplement: S2 Fig — Each panel is a recording. Fraction of variance explained by the classifier (fVE s2) is in the title of each panel (bootstrapped SE in parentheses). PC VE is the variance explained for the second principal component for each recording. (fVE cannot exceed PC VE.) Gray indicates that the classifier did not predict above chance levels (shuffle test). Inset shape shows the principal component (PC) loadings. Black is “amplitude” (Rec. 3, 8, 9, 11) and red is “sharpness/latency” (Rec. 1, 2, 4, 5, 6, 7, 10). Compare to S1 Fig, which shows the same plots for the first principal component. (PDF) [file pcbi.1006716.s002.pdf]

Classifier boundaries predicting weights on second PC

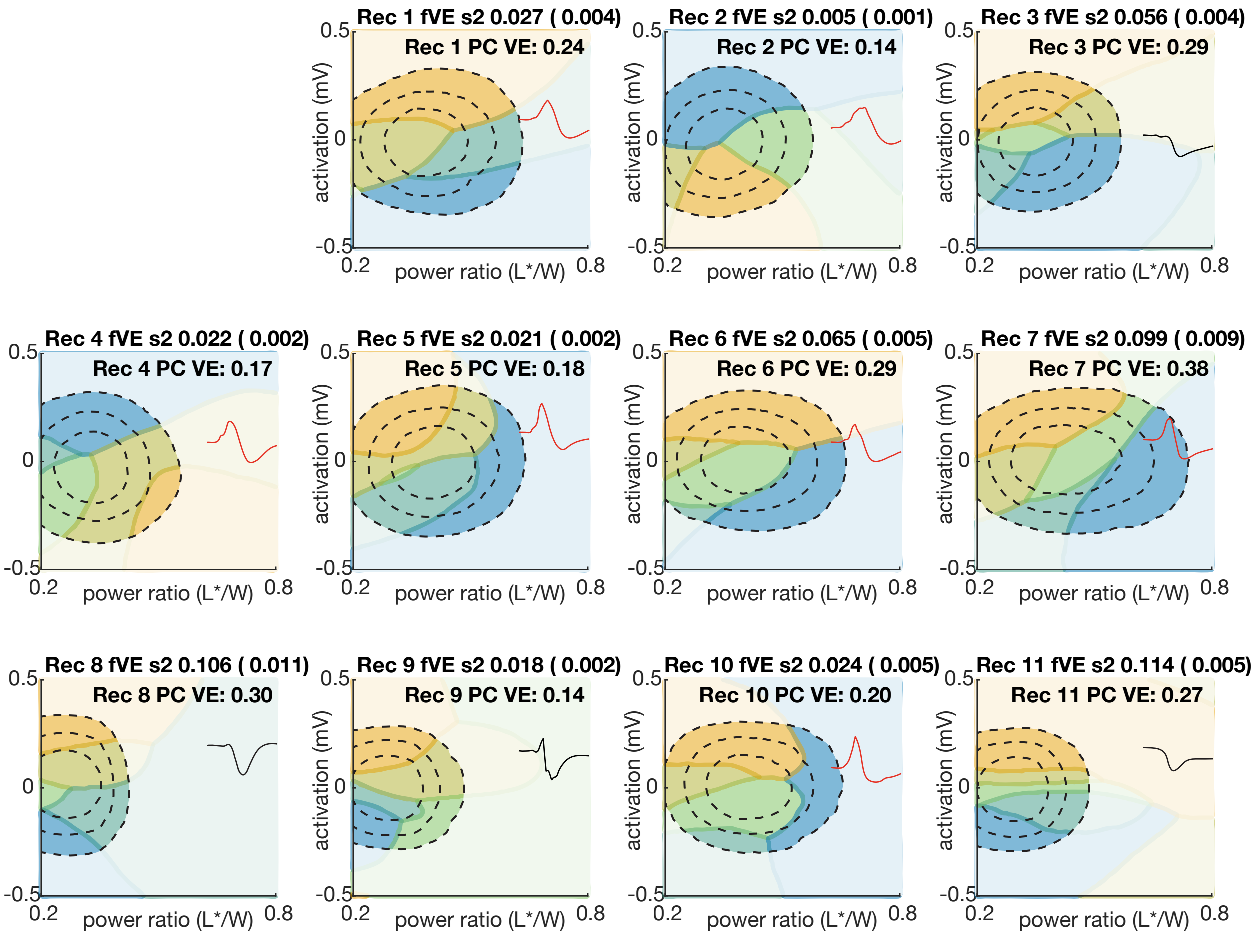

Supplemental Figure 2 (associated with Fig. 2): Classifier boundaries for the prediction of the weight onto the **second** principal component of the evoked responses for recordings 1 to 11. Each panel is a recording. Fraction of variance explained by the classifier (fVE s2) is in the title of each panel (bootstrapped SE in parentheses). PC VE is the variance explained for the second principal component for each recording. (fVE cannot exceed PC VE.) Gray indicates that the classifier did not predict above chance levels (shuffle test). Inset shape shows the principal component (PC) loadings. Black is “amplitude” (Rec. 3, 8, 9, 11) and red is “sharpness/latency” (Rec. 1, 2, 4, 5, 6, 7, 10). Compare to Supplemental Figure 1, which shows the same plots for the first principal component.
