# Supplementary material for: State-aware detection of sensory stimuli in the cortex of the awake mouse
Source: PLoS Comput Biol. 2019 May 31;15(5):e1006716. doi: 10.1371/journal.pcbi.1006716 (PMC6561583; doi:10.1371/journal.pcbi.1006716)
Supplement: S3 Fig — A-D: Same as Fig 4, but including a “multi-channel predictor,” constructed from the projection of pre-stimulus activity onto the top spatial modes in spontaneous LFP determined by PCA. (PDF) [file pcbi.1006716.s003.pdf]

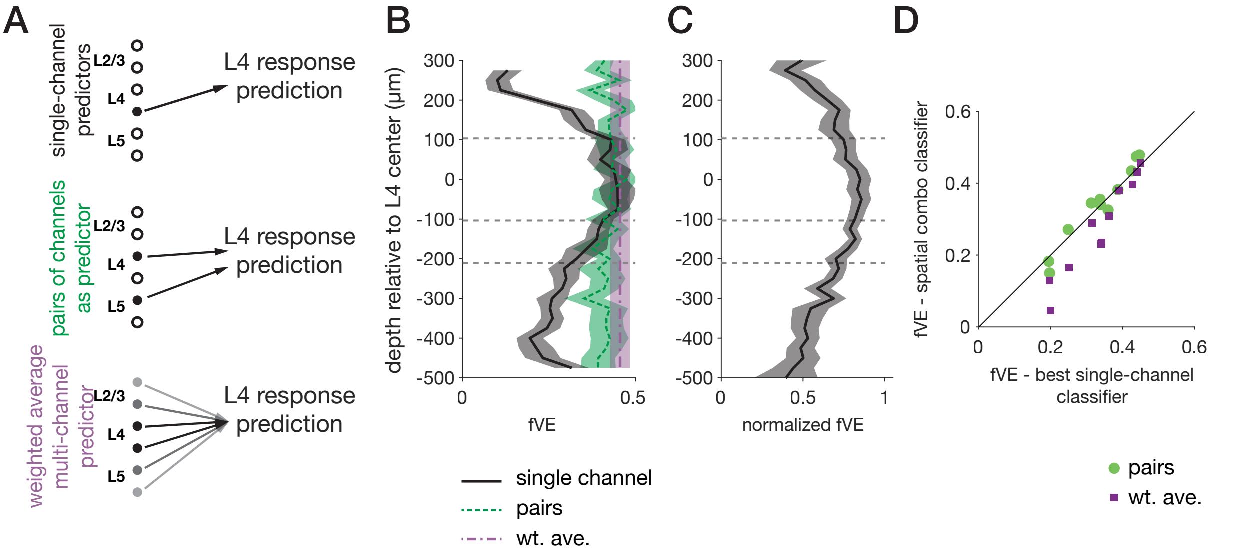

Supplemental Figure 3 (associated with Figure 4). Comparison of performance of different classifiers and more complex input transformation to the single-channel predictor.

A-D: Same as Fig. 4, but including a “multi-channel predictor,” constructed from the projection of pre-stimulus activity onto the top spatial modes in spontaneous LFP determined by PCA.
