# Supplementary material for: State-aware detection of sensory stimuli in the cortex of the awake mouse
Source: PLoS Comput Biol. 2019 May 31;15(5):e1006716. doi: 10.1371/journal.pcbi.1006716 (PMC6561583; doi:10.1371/journal.pcbi.1006716)
Supplement: S4 Fig — Column A: false alarms per s vs. threshold. Inset: fraction of spontaneous activity in each state. Column B: hit rates vs threshold. Column C: State-aware detection rates at fixed false alarm rate for combinations of state1 and state3 thresholds. Red contour is the detection rate with fixed threshold. Column D: Hit rate, conditioned on pre-stimulus state, in the state-blind and state-aware cases. (PDF) [file pcbi.1006716.s004.pdf]

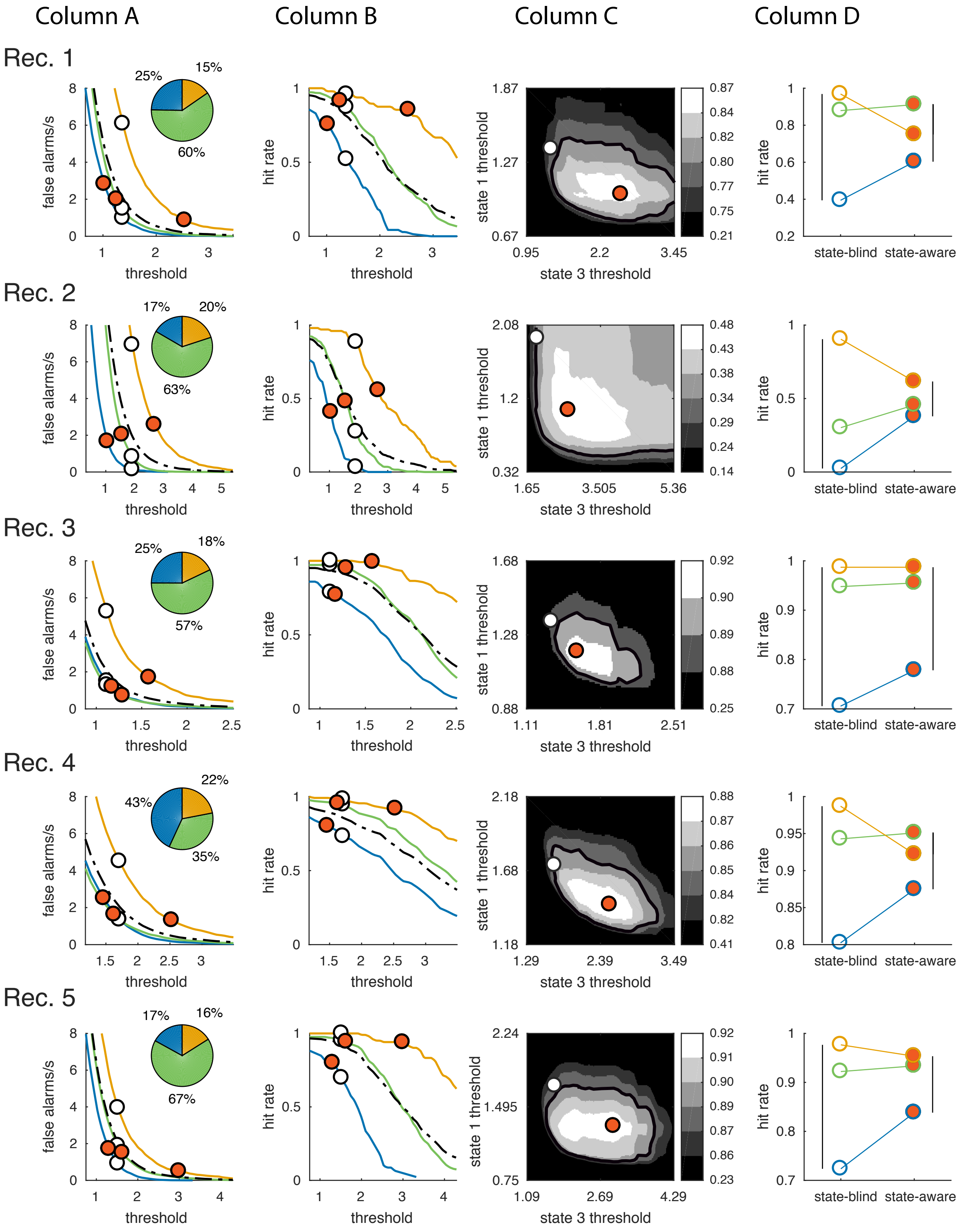

Supplemental Figure 4 (associated with Figure 6): Optimized thresholds for the state-aware observer. Panels A-D of Figure 6 for recordings 1 through 5. Column A: false alarms per s vs. threshold. Inset: fraction of spontaneous activity in each state. Column B: hit rates vs threshold. Column C: State-aware detection rates at fixed false alarm rate for combinations of state1 and state3 thresholds. Red contour is the detection rate with fixed threshold. Column D: Hit rate, conditioned on pre-stimulus state, in the state-blind and state-aware cases.
