# Supplementary material for: State-aware detection of sensory stimuli in the cortex of the awake mouse
Source: PLoS Comput Biol. 2019 May 31;15(5):e1006716. doi: 10.1371/journal.pcbi.1006716 (PMC6561583; doi:10.1371/journal.pcbi.1006716)
Supplement: S5 Fig — Column A: false alarms per s vs. threshold. Inset: fraction of spontaneous activity in each state. Column B: hit rates vs threshold. Column C: State-aware detection rates at fixed false alarm rate for combinations of state1 and state3 thresholds. Red contour is the detection rate with fixed threshold. Column D: Hit rate, conditioned on pre-stimulus state, in the state-blind and state-aware cases. Recording 6 is shown in Fig 6. (PDF) [file pcbi.1006716.s005.pdf]

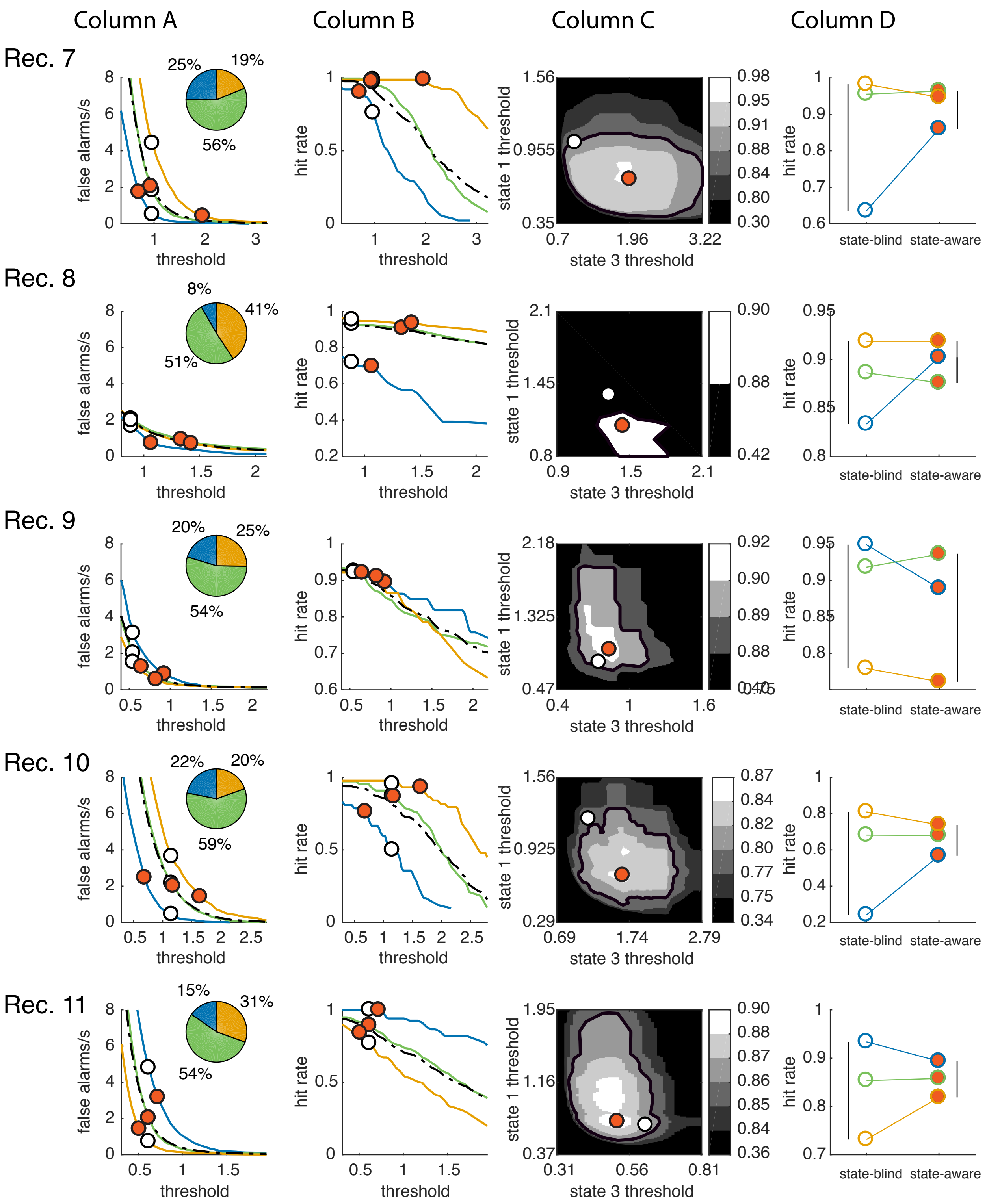

Supplemental Figure 5 (associated with Figure 6): Panels A-D of Figure 6 for recordings 7 through 11. See SFig 4 for caption. Recording 6 is shown in Figure 6.
